# Deep Brain Stimulation Reveals a Dissociation of Consummatory and Motivated Behaviour in the Medial and Lateral Nucleus Accumbens Shell of the Rat

**DOI:** 10.1371/journal.pone.0033455

**Published:** 2012-03-13

**Authors:** Geoffrey van der Plasse, Regina Schrama, Sebastiaan P. van Seters, Louk J. M. J. Vanderschuren, Herman G. M. Westenberg

**Affiliations:** 1 Department Psychiatry, Rudolf Magnus Institute of Neuroscience, University Medical Centre Utrecht, Utrecht, the Netherlands; 2 Department Neuroscience and Pharmacology, Rudolf Magnus Institute of Neuroscience, University Medical Centre Utrecht, Utrecht, the Netherlands; 3 Division of Behavioural Neuroscience, Department of Animals in Science and Society, Faculty of Veterinary Medicine, Utrecht University, Utrecht, the Netherlands; University of Chicago, United States of America

## Abstract

Following the successful application of deep brain stimulation (DBS) in the treatment of Parkinson's disease and promising results in clinical trials for obsessive compulsive disorder and major depression, DBS is currently being tested in small patient-populations with eating disorders and addiction. However, in spite of its potential use in a broad spectrum of disorders, the mechanisms of action of DBS remain largely unclear and optimal neural targets for stimulation in several disorders have yet to be established. Thus, there is a great need to examine site-specific effects of DBS on a behavioural level and to understand how DBS may modulate pathological behaviour. In view of the possible application of DBS in the treatment of disorders characterized by impaired processing of reward and motivation, like addiction and eating disorders, we examined the effect of DBS of the nucleus accumbens (NAcc) on food-directed behavior. Rats were implanted with bilateral stimulation electrodes in one of three anatomically and functionally distinct sub-areas of the NAcc: the core, lateral shell (lShell) and medial shell (mShell). Subsequently, we studied the effects of DBS on food consumption, and the motivational and appetitive properties of food. The data revealed a functional dissociation between the lShell and mShell. DBS of the lShell reduced motivation to respond for sucrose under a progressive ratio schedule of reinforcement, mShell DBS, however, profoundly and selectively increased the intake of chow. DBS of the NAcc core did not alter any form of food-directed behavior studied. DBS of neither structure affected sucrose preference. These data indicate that the intake of chow and the motivation to work for palatable food can independently be modulated by DBS of subregions of the NAcc shell. As such, these findings provide important leads for the possible future application of DBS as a treatment for eating disorders such as anorexia nervosa.

## Introduction

Deep brain stimulation (DBS) is increasingly used in the treatment of neurological and psychiatric diseases in patients that do not respond to conventional treatment [1]. Significant improvement of well-being (i.e. quality of life) has been observed in patients with Parkinsons's disease [2] and with obsessive compulsive disorder [3], [4]. As a result of these observations, DBS is now experimentally tested in small patient groups for the treatment of a broader group of disorders that include depression [5], [6], Tourette's syndrome [7], epilepsy [8], addiction [9] and eating disorders [10].

However, despite its broad therapeutic potential, the mechanisms of action of DBS are poorly understood and the application of DBS is very often based on case-studies rather than fundamental research. There is thus a great need to examine the effects of DBS on a behavioural level, which will allow for functional mapping of DBS targets and will clarify which aspects of behavioural dysfunction can be modified by DBS.

With respect to disorders that involve impaired processing of reward and motivation, like eating disorders and addiction, the behavioural effects of DBS of the ventral striatum (specifically the nucleus accumbens; NAcc) are of particular interest. Considering the critical role of the ventral striatum in the processing of reward-related information [11]–[15], it is reasonable to expect that disorders like addiction and eating disorders can benefit from DBS of ventral striatal subregions. It is, however, to a large degree unknown what the behavioural effects of NAcc DBS are in relation to reward processing and thus, which particular aspects of these disorders can be targeted with NAcc DBS.

The NAcc can be divided in a core and shell sub-region based on cytoarchitectonic criteria, afferent and efferent projections as well as function [11]–[17]. Although both core and shell are key structures in the processing of reward-related information and function as an interface between the limbic and motor systems, their specific projections indicate a functional differentiation [11]–[15]. Specific targeting of core and shell regions with DBS could thus provide a means to treat disorders characterized by dysfunctional processing of reward and motivation.

The NAcc core has been implicated in responding to reward-associated conditioned stimuli. Incorporation of these stimuli with motivational state within the core allows for the selection of appropriate motor responses and facilitation of instrumental conditioning [11], [13]–[15], [18]–[20]. Indeed, lesions or inactivation of the core attenuate the ability of conditioned stimuli to drive behavioral output [18], [21] and reduce the sensitivity to reward devaluation [22]. In contrast, the shell has been associated with unconditioned behaviours like food consumption (see below) and hedonic responses to unconditioned stimuli such as sucrose [23]. In addition, the shell has been suggested to drive the actual motoric output following response selection by the core [18], [22], but also in the influence of reward-associated sensory-specific cues on instrumental behaviour [21].

With respect to the role of the ventral striatum in consummatory behaviour and the possible application of DBS in the treatment of eating-disorders, like anorexia nervosa and binge eating, the functional specialisation within the NAcc shell that has been described in the modulation of feeding is of particular interest. Kelley and Swanson [24] observed a specific role of the medial wall of the shell (mShell) in the regulation of food intake. These authors showed that pharmacological inhibition of the mShell results in a large increase in the consumption of chow, whereas its activation decreases food intake, see also [25]. In contrast, pharmacological inactivation of the ventral/lateral shell regions (or core) did not affect feeding [13], [25]. Importantly, changes in consummatory behaviour following pharmacological inhibition with a GABA-A receptor agonist were subsequently shown not to affect operant responding for palatable food [26]. Follow-up studies by Stratford and Kelley [27] suggested that the enhanced feeding response observed following inactivation or lesions of the mShell is mediated by GABAergic projections from this area to the lateral hypothalamus. Based on these results and the possibility suggested by others that the effects of DBS resemble those of local lesions [2] (but see; discussion], specific targeting of the mShell could enhance food intake, and so be a potential target for the treatment of anorexia nervosa.

A small number of studies have so far examined differential effects of core and shell DBS. Sesia et al. [28] stimulated both areas in rats and showed that DBS of the core decreased impulsivity in a reaction-time task, whereas stimulation of the shell produced an increase. With regard to the application of DBS in the treatment of addiction, Vassoler and colleagues [29] have shown that DBS of the NAcc shell but not the core reduced reinstatement of extinguished cocaine, but not sucrose, seeking.

Together, the functional differentiation within the NAcc provides possible targets for DBS in the treatment of various psychiatric disorders. Therefore, a systematic characterization of the effects of DBS within these areas is of great importance. In view of the potential usefulness of DBS for the treatment of disorders characterized by dysfunctional reward and motivation, we examined the effects of DBS of three different subregions of the ventral striatum, i.e. the core, mShell and lateral shell (lShell) on food consumption, as well as the motivational and appetitive properties of food in rats. Based on the aforementioned literature we hypothesised that DBS will differentially affect the consumption of food and its appetitive/motivational properties by stimulation of the mShell and lShell/core, respectively. With respect to modulation of food intake, if DBS induces a local activation of neurons in the mShell a decreased consumption is expected. If, in contrast, DBS inactivates the stimulated area, food intake is likely to decrease.

## Materials and Methods

### Ethics Statement

All experiments were approved by the Animal Experimentation Committee of Utrecht University and were carried out in agreement with Dutch Laws (Wet op de Dierproeven, 1996) and European regulations (Guideline 86/609/EEC).

### Subjects

Subjects were male outbred Wistar rats (Charles River) weighing 200–225 g at arrival. Upon arrival, the animals were housed in groups of four in standard type IV macrolon cages. After surgery, the animals were kept in individual cages (25×25×35 cm). Food and water were available ad lib in the home cage for the duration of the experiment unless indicated otherwise. During the experiments that assessed the consumption of food (see below) the animals were kept under a 12-hour day/night cycle with lights on from 12:00 AM–12:00 PM. Animals tested on operant tasks and sucrose preference (see below) were kept under a reversed day/night schedule (lights on from 7:00 PM–7:00 AM).

### Surgery

Two weeks after arrival, the animals were implanted with bilateral stainless steel stimulation electrodes (PlasticsOne). Rats were anesthetised with ketamine hydrochloride (75.0 mg/kg, i.m.) and medotomidine (0.4 mg/kg, s.c.). The animals were subsequently mounted in a stereotaxic frame with the toothbar set at −2.5 mm. Electrodes were then placed in one of three target areas; NAcc core: angle 10° (in mm from bregma: A+0.3 L±1.6; V−7.7); NAcc lateral shell (lShell): angle 10° (in mm from bregma: A+1.2 L±2.8; V−8.3); NAcc medial shell (mShell): angle 17° (in mm from bregma: A+1.44 L±3.0; V−7.3). The electrodes were secured to the skull with dental cement and four cranial screws. Carprofen (5.0 mg/kg, s.c.), was given directly following surgery and on the two days after for postoperative pain relief.

### Apparatus

Tests of food consumption and operant behaviour were performed in operant chambers (29.2×24.1×21 cm; Med Associates) equipped with two retractable levers, a food-receptacle with infra-red nose-poke detection, house-light and grid floor. An electrically shielded commutator (Plastics One) attached to a counter-balanced arm (Med Associates) was placed above the box, allowing for free movement during testing. Stimulation cables were protected by a wire-mesh. Operant chambers were controlled by a computer running Med-PC ™ software (Med Associates). A sound-attenuating chamber enclosed the operant chambers.

For the measurement of food-intake, a glass container with normal lab chow (SDS) was placed inside the operant chambers. Apart from a house-light, there were no functionally active components in the chamber at the time of testing. To prevent possible interaction effects between DBS-induced changes in fluid intake and food intake, no water was available during testing.

Sucrose preference was tested in Perspex cages (26×26×35 cm) onto which two bottles were mounted, one containing water, the other a 1% sucrose solution. To prevent possible interaction effects between DBS-induced changes in fluid intake and food intake, no food was available during testing.

A radio continuously played in both the animal facility and test rooms to attenuate interference from background noise.

### Behavioural procedures

#### Food intake

Two weeks following surgery, the rats were subjected to a minimum of five training sessions, during which they were placed in the test chambers and given access to a bowl of pre-weighed chow, identical to the food available in their home-cage. The animals remained in the test chamber for 1 hour (the second-to-last hour of the light-phase); food was weighed after 30 minutes and at the end of the session (1 hour). Following this period, the animals were returned to their home cages. Following this initial exposure to the operant chamber, the animals were habituated to the stimulation procedure. To this end, the rats were attached to the stimulation cables without passing a current. Testing commenced as soon as animals showed stable intake of food while attached to the stimulation cables (>0.5 gr/session; this usually took 3–5 sessions). First, two baseline sessions were performed, during which animals were tested as during habituation but without connection to the stimulation cables. Following these baseline sessions, the animals were subjected to a test session in which they were attached to the stimulation cables and 1 of 3 stimulation intensities was applied (10 µA, 50 µA or 100 µA). To exclude possible effects of the attachment to the stimulation cables, animals were also subjected to a test session in which they were attached to the cables but no current was applied (‘0 µA’ sham control). All animals received tests with all four stimulation intensities according to a latin-square design. Stimulation was given for the entire 1-hour period of testing, without interruption. Stimulation sessions were separated by at least 2 baseline sessions to avoid possible residual effects of stimulation.

#### Operant behaviour

The animals were tested under progressive ratio and fixed ratio 1 schedules of reinforcement. The progressive ratio schedule is commonly used to assess the motivation to obtain reward [30], [31]. For comparison, we also included a low-effort, fixed-ratio 1 schedule of reinforcement.

#### Fixed ratio 1 schedule

Two weeks following surgery, the animals were trained, in 20 minute shaping sessions, to press a lever for sucrose pellets (45 mg, formula F, Research Diets, New Brunswick, NJ, USA). During this shaping phase, a single lever was presented in each trial, with a 15 sec inter-trial interval (ITI). Pressing the lever resulted in illumination of the cue-light above the lever, reward delivery and retraction of the lever. Following initial training, the animals were habituated to the stimulation cables for a minimum of 5 sessions. During this time, second, inactive, lever was introduced and session duration was increased to one hour. Responding on the inactive lever had no programmed consequences. As soon as animals showed stable task performance (≤10% deviation in number of rewards over 3 consecutive days), the animals were subjected to a test session in which 1 of 3 stimulation intensities was applied according to a latin-square design (10 µA, 50 µA or 100 µA). In contrast to the food-intake experiment (see above) no ‘0 µA’ control was included because animals in this experiment were attached to the stimulation cables throughout the experiment. All animals received tests with all stimulation intensities. Stimulation was given for the entire 1-hour period of testing. Stimulation sessions were separated by at least three baseline session to avoid possible residual effects of stimulation. During baseline sessions, the animals were attached to stimulation cables, but not stimulated.

#### Progressive ratio schedule

Initially, the animals were trained to press a lever for a sucrose reward as described above for the fixed ratio 1 schedule. Identical to the procedure for the fixed ratio 1 experiment, the animals were attached to the stimulation cables for habituation following the initial shaping phase. Over the course of 4–7 sessions, the response requirement was increased from a fixed ratio of 1 to 3 responses and subsequently to 5 responses. Next, the animals were trained to respond under a progressive ratio schedule, in which each subsequent reward delivery (2 sucrose pellets) required more lever-presses (i.e. 1, 2, 4, 6, 9, 12, 15, 20, 25, 32, 40, 50, 62, 77, 95, 118, 145, 178, 219, 268, 328, 402, 492, 603, 737, 901) [31]. Sessions ended whenever an animal failed to obtain a reward within 30 min after the last reward; test duration thus varied depending on the animals' performance. Upon reward delivery, the levers were retracted and the cue light was turned on. The inter-trial interval was set at 30 s. A second, inactive, lever was present in the chamber; pressing this lever was without programmed consequences. The procedure for stimulation sessions and criterion for stable performance was similar to that of the fixed ratio 1 schedule (see above). Stimulation was given for the entire period of testing.

#### Sucrose preference

Animals that were previously trained to respond for sucrose pellets under a fixed-ratio 1 schedule were subsequently subjected to a sucrose-preference test. This allowed us to establish preference in animals that were familiar with sucrose as a reward. After the fixed ratio 1 experiment, the animals were given access to a 1% sucrose solution in their home cage, for at least 5 days. In addition to food and water, a sucrose solution was available ad libitum until testing. On test days, animals were deprived of water and sucrose solution for 4 hours prior to testing. During the subsequent test phase, water and the 1% sucrose solution were freely available. On stimulation days, the animals were connected to the stimulation cables and received, according to a latin-square design, one of the stimulation intensities (‘0 µA’ or sham stimulation, 10 µA, 50 µA or 100 µA), similar to the procedure for food intake. Stimulation was given for the entire 1-hour period of testing.

### General

For the food intake experiment animals were tested during the light phase. Testing during this period, when the consumption of food is moderate, allowed for DBS-induced increases as well as decreased of food intake. Testing during the dark phase, when food intake is high, could potentially obscure DBS-induced increases in consumption of chow. In contrast, operant tests and sucrose preference was performed during the dark phase when animals are in their active period.

### Behavioural measures

#### Food intake

Chow intake was measured by weighing the glass container before and after the 1 hour session. To examine the potential time-dependent effect of DBS, the container was also weighed halfway through the session. Food intake was then compared between the first and second half of the session. Spillage of chow was measured by weighing food that fell through the grid or was left on the grid floor after cessation of the experiment.

#### Operant behaviour

The variable taken as measure of performance was the total number of rewards obtained, and the number of presses on the active lever. Responses on the inactive lever and the number of entries into the food dispenser (i.e. nose-pokes) were taken as a measure of general activity. To assess possible changes in response discrimination (i.e. responses on the active- vs. inactive levers), ratios of these parameters were also analysed.

#### Sucrose preference

Water and sucrose intake were measured by weighing the bottles of water and sucrose before and after a 1 hour session. Preference was calculated as the ratio between water and sucrose (1%) intake by the following formula: (sucrose intake (g)/(sucrose intake (g)+water intake (g)) [32].

### Stimulation

Stimulation was performed with a digital stimulator (World Precision Instruments, DS8000) and stimulus isolator (World Precision Instruments, DLS100). During stimulation experiments, the electrode implants (dual stainless steel electrodes with 300 µm exposed tip; PlasticsOne) were attached to stimulation cables which were connected to stimulation equipment through an electrically shielded commutator (Bilaney Consultants). Stimulation parameters were as follows; biphasic square pulses, 60 µS duration, 200 µS ‘zero’ time, frequency 130 Hz.

### Data analysis

#### Food intake

Total food intake (g) during DBS (0 µA,10 µA, 50 µA and 100 µA) was compared with overall average baseline food intake over the 2 days prior to the stimulation sessions (measured at the same time of day, but without connection to the stimulation cables). A one-way ANOVA with a Student-Newman-Keuls (SNK) post-hoc test was used to analyse differences between stimulation intensities. If stimulation was observed to affect food intake, an additional paired-samples t-test was performed between the first and second half of the session. This analysis was performed to explore possible time-dependent effects of DBS. A comparison (one-way ANOVA with SNK) between baseline food intake of the three experimental groups (ie. core, lShell and mShell) was made to exclude pre-existing differences in intake (possible induced by, location specific, effects of electrode placement) prior to testing. In a similar fashion, in case an effect of stimulation was observed, the data of the 2-day base-line preceding the stimulation day was compared between stimulation intensities within a target area. This analysis was performed to exclude order effects.

#### Operant behaviour

The number of rewards that were obtained under a fixed ratio 1 and progressive ratio schedule during DBS (10 µA, 50 µA and 100 µA) was compared with the overall average baseline performance over 3 days prior to the stimulation sessions. A one-way ANOVA with a SNK post-hoc test was used to analyse differences between stimulation intensities. The number of responses that were made on the active lever in both tasks was analysed in a similar fashion. Similar to the analyses of the food intake, potential pre-existing differences in base-line performance were analyzed (one-way ANOVA with SNK) for all three groups (ie. core, lShell, mShell). Likewise, in case an effect of stimulation was observed, the data of the 3-day base-line preceding the stimulation day were compared between stimulation intensities within a target area. This analysis was performed to exclude order effects. To compensate for individual differences between animals, the data were normalized relative to base-line values. Similar analyses were performed for responses on the inactive lever, for nose-pokes as well as for the ratio of active to inactive lever responses.

#### Sucrose preference

Effects of stimulation (0 µA,10 µA, 50 µA and 100 µA) on sucrose preference, as well as on the absolute intake of water and sucrose solution were compared (one-way ANOVA with SNK) with overall average baseline intake over the 2 days prior to the stimulation sessions (measured at the same time of day, but without connection to the stimulation cables). Similar to the analyses of the food intake, potential pre-existing differences in base-line sucrose/water ratios were analysed for all three brain areas (one-way ANOVA with SNK). In case an effect of stimulation was observed, the data of the 3-day base-line preceding the stimulation day were compared between stimulation intensities within a target area. This analysis was performed to exclude order effects.

### Histological examination

After completion of the experiments, the rats were killed by inhalation of a mixture of CO_2_/O_2_ (70/30) followed by 100% CO_2_. The brains were rapidly taken out of the skull and frozen at −80°C. Coronal (40 µm) sections were cut on a cryostat, stained with thionine and examined with a microscope to determine precise location of the electrodes.

## Results

### Histological analysis

**Figure 1:**
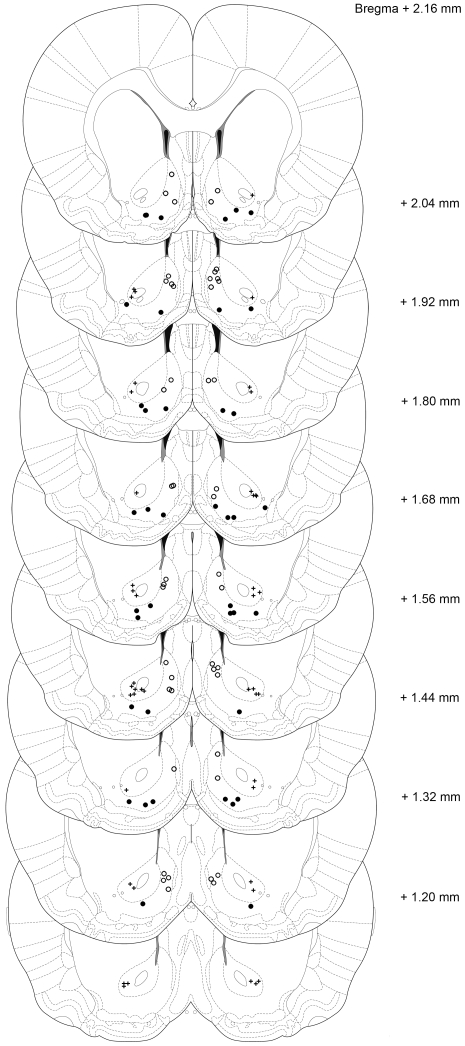
Localization of the electrode-tips of all animals. The plus symbols indicate endpoints of electrodes placed in the NAcc core, closed circles indicate endpoints of electrodes placed in the lateral shell, and open circles indicate endpoints of electrodes placed in the medial shell. Animals with electrodes placed outside the target area were not included in the analysis (adapted from; [33]).

Brain sections were examined for placement of the stimulation electrodes. Figure 1 shows electrode placement for each of the three areas of interest (i.e. NAcc core, lShell and mShell). Only the data from animals in which both electrodes were placed in the target area were included in the analysis. Following histological analysis, the experimental groups contained the following number of animals (number of excluded animals between brackets): Fixed ratio 1 schedule and sucrose preference: core n = 7(3), mShell n = 8(2), lShell n = 7(1); progressive ratio schedule: core = 7(4), mShell n = 7(3), lShell n = 6(1); Food intake: core n = 8(2), mShell n = 8(2), lShell n = 7(3).

### Food Intake

**Figure 2:**
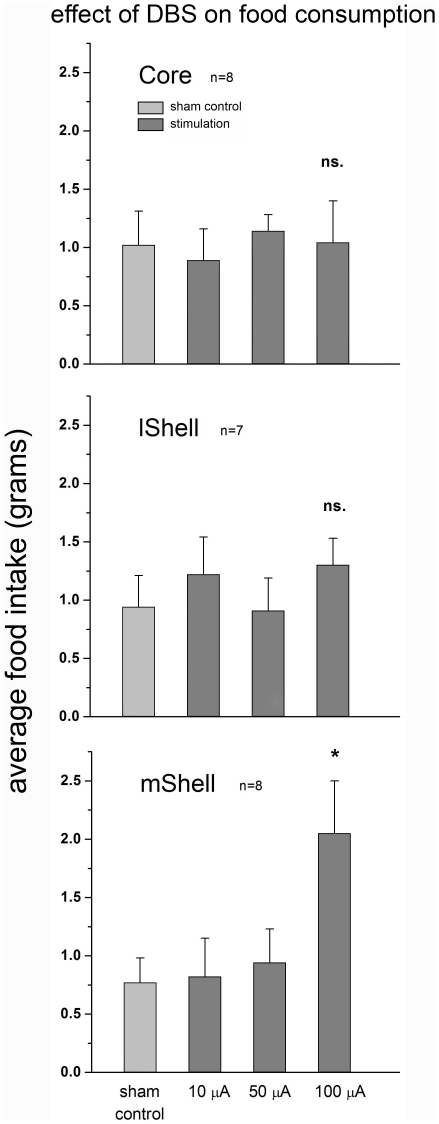
Effect of DBS on the consumption of food. The graph represents mean food intake in grams (+SEM) (y-axis). Intensity of stimulation is depicted on the x-axis. Stimulation of the core (top graph) or lShell (middle) did not affect food intake. Stimulation of the mShell (bottom) at the highest intensity (100 µA) significantly increased food intake. Stimulation at lower intensities did not affect food consumption in any target area.

Group comparison between sham-stimulation and base-line (without connection to the stimulation cables) showed no significant difference in any of the stimulation areas (F-value between 0.02 and 0.332, P>0.57; data not shown). This indicates that connection to the stimulation cables (without current) did not affect food intake. Similarly, there was no difference in baseline intake (without stimulation cables) between the DBS sites (F2,20 = 0.26, P = 0.77). The results of the food intake experiment are shown in Figure 2.

#### Core

There was no overall effect of stimulation intensity on total food intake (F4,35 = 0.24, P = 0.92). Average baseline food intake did not differ from intake following stimulation (Figure 2).

#### Lateral Shell

There was no overall effect of stimulation intensity on total food intake. (F4,30 = 0.51, P = 0.73). Average base-line food intake did not differ from intake following stimulation (Figure 2).

#### Medial Shell

A one-way ANOVA indicated that there was an overall effect of stimulation on total food intake (F4,32 = 3.44, P = 0.02). The post-hoc test showed that only DBS at the highest intensity (100 µA) significantly increased food intake compared to all other groups (base-line average 0.81±0.1 gr, 100 µA DBS average 2.05±0.45 gr, P<0.05). A comparison between food intake during the first half hour of stimulation and the second half hour indicated that there was no difference (1.15±0.39 gr vs. 0.90±0.27 gr; F1,14 = 0.27, P = 0.61). An additional one-way ANOVA over the base-line data of each of the stimulation conditions was performed to exclude order effects. No differences were found (F4,32 = 0.03, P = 1.00), showing that DBS during measurements does not affect performance on later sessions.

### Operant behaviour

**Figure 3:**
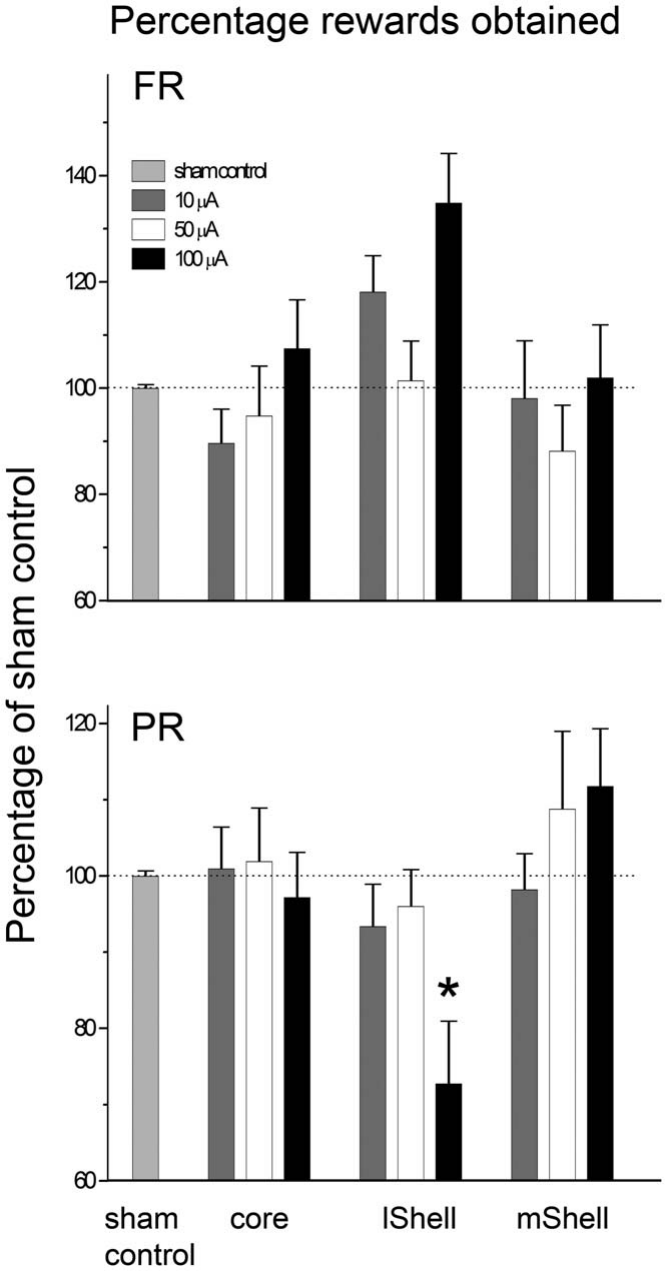
Effect of DBS on operant responding for sucrose. The graph represents the mean number of obtained rewards (+SEM) as a percentage of those obtained during sham stimulation (y-axis). Stimulation areas are depicted on the x-axis. Stimulation of the NAcc (core, lShell or mShell) did not significantly affect responding for sucrose (top) but there was a strong trend towards a significant increase in lShell stimulated animals. Responding under a progressive ratio schedule for sucrose (bottom) was not affected by DBS of the core or mShell. Stimulation of the lShell at the highest intensity (100 µA) significantly decreased responding. Stimulation at lower intensities did not affect performance in either task, in any target area.

Results of both the fixed ratio 1 and progressive ratio schedule are depicted in Figure 3, average values for active- and inactive lever presses are depicted in table 1. To exclude possible pre-existing differences in performance, base-line values were compared between the stimulation intensities (one-way ANOVA with SNK). No significant differences were observed, for either the fixed ratio 1- or progressive ratio experiment (F-value between 0.91 and 1.35, P>0.29; data not shown). To compensate for variation in performance between animals, the data were normalized relative to base-line values. Variation in the number of lever presses was observed during stimulation and base-line sessions, as well as within stimulation areas. This indicates that individual variation was not specific to any condition.

**Table 1:** Average number of lever presses per condition.

			base-line	10 µA	base-line	50 µA	base-line	100 µA
**FR 1**	core	active	68.57 (8.99)	63.14 (11.82)	82.90 (11.22)	75.57 (8.41)	75.57 (8.41)	82.43 (14.07)
		inactive	2.76 (1.18)	2.14 (0.96)	5.52 (2.20)	1.00 (0.49)	3.86 (1.85)	1.00 (0.38)
	lShell	active	97.05 (13.53)	111.71 (15.96)	89.43 (9.42)	93.14 (15.34)	64.67 (13.60)	80.57 (16.11)
		inactive	7.00 (2.85)	7.71 (4.75)	3.38 (0.77)	2.14 (1.10)	2.33 (0.68)	1.86 (1.03)
	mShell	active	69.25 (5.51)	70.00 (10.07)	81.04 (9.96)	67.00 (10.00)	76.75 (10.53)	77.50 (10.59)
		inactive	23.17 (13.79)	2.75 (1.52)	2.00 (0.59)	0.625 (0.50)	1.92 (0.68)	3.38 (1.10)
**PR**	core	active	253.71 (48.12)	221.43 (19.74)	251.62 (22.03)	257.43 (37.57)	225.43 (33.19)	226.57 (49.65)
		inactive	1.33 (0.61)	0.57 (0.43)	7.00 (2.25)	6.14 (2.44)	4.43 (1.51)	1.57 (0.87)
	lShell	active	488.39 (92.70)	482.33 (103.38)	284.33 (48.83)	322.5 (77.85)	410.11 (61.89)	179.167 (51.50)
		inactive	14.39 (7.41)	7.83 (4.25)	10.89 (3.67)	4.83 (1.83)	6.00 (1.90)	3.33 (2.95)
	mShell	active	163.90 (26.34)	155.86 (24.35)	128.57 (26.38)	148.43 (38.88)	149.48 (43.72)	206.71 (63.98)
		inactive	3.38 (1.25)	3.43 (1.25)	2.29 (1.26)	0.00 (0.00)	1.76 (0.63)	2.71 (0.94)

The average (with SEM in parentheses) number of active- and inactive lever presses per condition with base-line values for each stimulation intensity. FR 1, Fixed ratio 1, PR, progressive ratio.

#### General activity

To assess effects of DBS on general activity, we compared nose-poke activity (average FR1 (SEM) 9.39±0.72<11.84±0.62; average PR (SEM) 209.06±20.35<592.52±50.47) and activity at the inactive lever (average FR1 (SEM) 2.29±0.37<3.94±.71; average PR (SEM) 2.48±0.45<9.83±1.87). In none of the groups in any of the experimental conditions did DBS affect these measures (P>0.10, P<0.98). Ratios of active/inactive lever presses were not significantly different either (P>0.20). These data indicate that changes in behaviour are not due to a general change in task-related activity.

#### Core

Fixed ratio 1: There was no overall effect of stimulation on number of obtained rewards (F3,24 = 0.67, P = 0.58) or number of active lever responses (F3,24 = 0.64, P = 0.58). Progressive ratio: There was no overall effect of stimulation on obtained the number of rewards (F3,24 = 0.13, P = 0.94) or active lever presses (F3,24 = 0.82, P = 0.97).

#### Lateral Shell

Fixed ratio 1: There was an overall effect of stimulation intensity on number of obtained rewards (F3,24 = 3.35, P = 0.04). The post hoc test indicated that there was a near-significant increase in the number of obtained rewards in the 100 µA group when compared to the other stimulation intensities (P = 0.051). Similarly, a trend towards a group effect was observed for the number of responses on the active lever (F3,24 = 2.99, P = 0.051). Progressive ratio: There was an overall effect of stimulation on the number of obtained rewards (F3,20 = 4.44, P = 0.02). The post hoc test showed that DBS at the highest intensity (100 µA) significantly decreased the number of rewards compared to all other groups (72.75% of base-line, P<0.05). Analysis of the number of responses on the active lever corroborated these findings, showing a significant group effect (F3,20 = 3.758, P = 0.027). Post-hoc analysis indicated a significant reduction of active lever pressing in the 100 µA DBS group (P<0.05). An additional one-way ANOVA over the base-line data of each of the stimulation conditions was performed to exclude order effects. No differences were found (F2,15 = 0.72, P = 0.50), showing that DBS during measurements does not affect performance on later sessions.

#### Medial Shell

Fixed ratio 1: There was no overall effect of stimulation on number of obtained rewards (F3,28 = 0.41, P = 0.75) or active lever responses (F3,28 = 0.53, P = 0.67). Progressive ratio: There was no overall effect of stimulation on the number of rewards (F3,24 = 0.80, P = 0.51) or active lever responses (F3,24 = 1.133, P = 0.36).

### Sucrose preference

Group comparison between sham-stimulation and base-line sucrose intake (without stimulation cables) revealed that there was no significant difference in any of the stimulation areas (F-value between 0.20 and 2.57, P>0.14). Similarly, no difference was found between sham-stimulation and base-line water intake in any of the areas (without stimulation cables) (F-value between 0.34 and 4.71, P>0.05). There was no difference in baseline intake (without stimulation cables) between the DBS sites for sucrose or water (resp. F = 0.57, P = 0.58; F = 1.44, P = 0.26).

**Figure 4:**
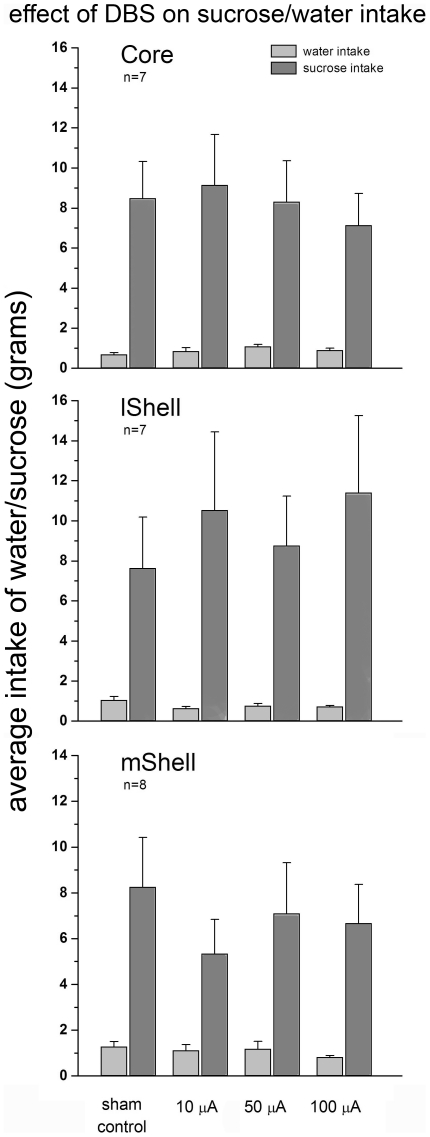
Effect of DBS on sucrose preference. The graph represents mean preference ratio's (±SEM) for all target groups (core, lshell, mshell) (y-axis). Stimulation-intensities are depicted on the x-axis. Base-line preference was significantly different from chance-levels (50% indicated by dotted-line), but was not affected by stimulation, for any group.

Average base-line preference ratios (without stimulation cables) for core, lShell and mShell indicate a preference for sucrose over water in all groups (resp., 0.89±0.02, 0.92±0.02 and 0.84±0.04; see figure 4), these values were not significantly different between stimulation sites (F2,21 = 1.70, P = 0.21).

#### Core

**Figure 5:**
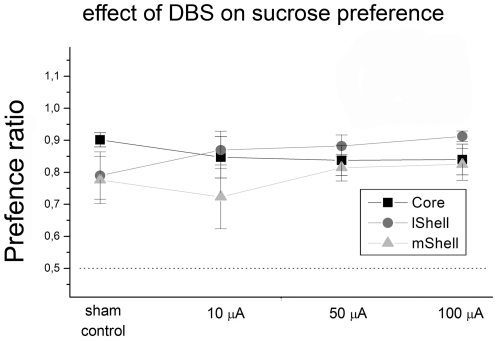
Effect of DBS on sucrose/water intake. The graph represents the mean intake of water and sucrose (1%) in grams (+SEM) (y-axis). Stimulation-intensities are depicted on the x-axis. Stimulation of the NAcc core (top), lShell (middle) or mShell (bottom) did not affect intake of either water or sucrose solution.

There was no overall effect of stimulation intensity on sucrose preference (F4,30 = 0.44, P = 0.78). Analyses of the intake of sucrose and water revealed no changes in consumption of either solution following DBS (resp. F4,30 = 1.41, P = 0.26; F4,30 = 0.20, P = 0.92), see figure 5.

#### Lateral Shell

There was no overall effect of stimulation on sucrose preference (F4,30 = 1.27, P = 0.30). Analyses of the intake of sucrose and water revealed no changes in consumption of either solution following DBS (resp. F4,30 = 1.63, P = 0.19; F4,30 = 0.50, P = 0.74), see figure 5.

#### Medial Shell

There was no overall effect of stimulation on sucrose preference (F4,35 = 0.50, P = 0.74). Analyses of the intake of sucrose and water revealed no changes in consumption of either solution following DBS (resp. F4,35 = 0.63, P = 0.65; F4,35 = 1.10, P = 0.37), see figure 5. Despite high preference ratios, individual preference scores (range: 0.42–0.98) suggest that changes in preference following DBS would have been detectable and are not obscured by ceiling effects.

## Discussion

In the present study we investigated the effects of DBS of subregions of the NAcc on food-directed behaviour in rats and explored potential anatomical and therapeutic DBS targets for the treatment of eating disorders. In keeping with the functional and anatomical diversity within the NAcc, our results show a dissociation between the effects of DBS of NAcc subregions on operant responding and consummatory behaviour. Whereas DBS of the mShell resulted in an increase in food intake without affecting operant responding for food, DBS of the lShell altered responding for sucrose, but did not change the consumption of freely available chow. Stimulation of the NAcc core did not affect either of these measures, and sucrose preference was not altered by DBS of the NAcc core, lShell or mShell. The double dissociation of the effects of lShell and mShell DBS on reward-directed behaviour is particularly relevant with respect to the clinical application of DBS for the treatment of disorders that affect motivational, or consummatory processes, like eating disorders and addiction.

We found that DBS of the mShell increased food intake up to 250% of baseline values. This effect was specific to this area as stimulation of the NAcc core or lShell did not affect food intake. This anatomically specific effect is in keeping with literature on the regulation of eating that has implicated the mShell as an important modulatory brain area in the regulation of food consumption. It is well established that inactivation of the mShell, either through stimulation of GABA receptors or antagonism of AMPA/kainate receptors, results in a robust increase in feeding in non-deprived animals without affecting water intake or locomotion [24], [25], [34]. Blockade of GABA receptors within the mShell, on the other hand, reduces deprivation-induced food intake [35]. The effect of DBS of the mShell was behaviourally specific, as DBS of the mShell did not affect operant responding for sucrose, or sucrose preference. Such a functional specificity of mShell neurons is consistent with earlier observations by Zhang and colleagues [26] who showed that infusions of the GABA-A receptor agonist muscimol into the mShell did not affect responding for sucrose under a progressive ratio schedule of reinforcement, but see [36]. Likewise, a recent DBS study by Vassoler et al. [29] showed that DBS of the mShell did not affect reinstatement of extinguished responding for sucrose. With respect to the lack of effect on sucrose preference and intake, pharmacological inactivation of the mShell has previously been shown to increase intake of a 5% sucrose-solution, over a cumulative period of 3 hours [34]. However, intake of sucrose solution was not significantly increased during the first hour of testing. The lower concentration of sucrose used in the present experiment (i.e. 1%) may have further reduced any possible effects of DBS of the mShell on sucrose intake. Together, these data suggest that the mShell may be a DBS target for specific augmentation of food intake without affecting the incentive motivational or general appetitive properties of food.

With respect to such a specific action, the mShell has been shown to embody ‘hedonic hotspots’, and ‘defensive’ centres, as well as ‘appetitive’ centres, in which converging glutamatergic, dopaminergic, opioid and GABAergic neurotransmission can modulate positive and negative emotional responses [37]. Histological analysis of electrode placements revealed that the mShell was targeted along a rostral-caudal axis that covers both ‘appetitive-’, and ‘defensive’ centres and hotspots, making it likely that DBS affected both types of emotional processes. In light of the absence of an effect on operant responding for sucrose and sucrose preference, suggesting that mShell DBS did not interfere with appetitive and incentive motivational responses to food, these data suggest that DBS preferably targets a sub-population of neurons or signal transduction process that drives food intake. Indeed, if hedonic hotspots within the mShell were also affected by DBS, this would likely have been reflected in altered operant responding for sucrose and sucrose preference [13]. Interestingly, it was recently reported that feeding behaviour and food hedonics are modulated by dissociable mechanisms in the mShell [38]. Thus, infusion of an AMPA/kainate receptor antagonist into the mShell increased feeding, but failed to alter orofacial affective (hedonic) responses to sucrose. In contrast, infusion of the GABA-A receptor agonist muscimol into the mShell enhanced both feeding and hedonic responses to sucrose. In the present study, orofacial responses to sucrose were not measured. Since the hedonic properties of food are only one (but important) factor that drives operant responding for food and sucrose preference, it can not be inferred from the present data whether DBS of the NAcc alters the hedonic properties of food.

Based on both preclinical and clinical work, DBS has been suggested to induce a lesion-like effect in the target area [2]. There is, however, ample evidence that this is an oversimplification. Although the effects of DBS in Parkinson's disease patients are often described as immediate, other patient groups (e.g. obsessive compulsive disorder patients) show only gradual amelioration of symptoms after prolonged stimulation, suggesting that neuroplastic changes, at least in part, underlie the behavioural effects of DBS [39]. Although, similar to mShell DBS, both lesions and pharmacological inactivation of the mShell have been shown to increase food intake [24], [25], [34], [40], the absence of an effect of mShell DBS on sucrose preference argues against a straightforward lesion- or inactivation-like effect of DBS [34], [41]. In addition, DBS of the NAcc core did not affect general motor activity whereas lesions of this area have been shown to induce hyperactivity [18]. Rather, the findings reported here echo those described by Faure et al. [38] for antagonism of AMPA/kainate receptors in the mShell, suggesting that DBS might specifically reduce glutamatergic drive into the mShell. Such selectivity of DBS on a specific neurochemical input has not been described so far, but it is clear that the physiological effects of DBS depend heavily on the neural element that is affected (e.g. cell-body or axon, cell-size and degree of myelinisation) and thus the target areas as well as its afferents and efferents. That is, DBS can ‘activate’ axons both orthodromically as well as antidromically [42]. Given the differential distribution of GABA and AMPA/kainate receptors on mShell neurons, a differential effect of mShell DBS on glutamatergic neurotransmission is not unlikely [38].

The present findings are particularly relevant with regard to the possible clinical application of mShell DBS for the treatment of eating disorders like anorexia nervosa, given the behavioural specificity of the effects. Although our data show sustained food intake during mShell DBS, further experimentation is needed to assess long-term effects of DBS on food intake and body weight.

In contrast to the increase in consummatory behaviour observed after mShell DBS, stimulation of the lShell affected operant responding for sucrose. Following stimulation of the lShell, responding for sucrose under a progressive ratio schedule decreased significantly, but there was a strong trend towards increased responding for sucrose under a fixed ratio 1 schedule of reinforcement, both in terms of obtained rewards as well as number of responses on the active lever. Neither food, or sucrose, intake nor sucrose preference were affected by DBS of the lShell, suggesting that the altered motivation to respond for sucrose does not reflect DBS-induced alterations in food consumption. In addition, since operant performance under the progressive ratio schedule decreased whereas it increased under the fixed ratio 1 schedule, it is not likely that the changes in responding for food are secondary to general motoric output.

In contrast to the well-documented role of the mShell in the modulation of food-directed behaviour, the function of the lShell in reward and motivation has not been investigated in great detail. A recent study suggested that neuroplasticity in dopaminergic projections to the lShell is particularly related to emotional salience [43], which, in turn, could modulate food-directed behaviour. The present data are reminiscent of those observed after administration of dopamine receptor antagonists during psychostimulant self-administration. In those experiments, reducing the reinforcing properties of cocaine with a dopamine receptor antagonist led to a decrease in breakpoint under a progressive ratio schedule of reinforcement, which coincided with increased responding under a fixed-ratio schedule of reinforcement, as if to compensate for the reduced subjective effects of the drug [44]–[46]. Future experiments should elucidate whether altered appetitive, hedonic or reinforcing properties of sucrose underlies the effects of lShell DBS on operant responding for sucrose.

Taken together, the lack of effect observed following core stimulation and the complex effects of lShell stimulation on operant responding for food does not provide a straightforward lead for the applicability of DBS of these areas for eating disorders. In order to better understand the behavioural and neural background of its effects on reward processes, further experiments are required.

### Overall conclusion

Our data indicate that DBS of the rat NAcc alters motivational and consummatory processes in an anatomically and behaviourally specific manner. Importantly, the selective functional effects of NAcc mShell DBS on feeding provide a possible target for the treatment of eating disorders.
